# A Mouse Model of Harlequin Ichthyosis Delineates a Key Role for Abca12 in Lipid Homeostasis

**DOI:** 10.1371/journal.pgen.1000192

**Published:** 2008-09-19

**Authors:** Ian Smyth, Douglas F. Hacking, Adrienne A. Hilton, Nigora Mukhamedova, Peter J. Meikle, Sarah Ellis, Keith Slattery, Janelle E. Collinge, Carolyn A. de Graaf, Melanie Bahlo, Dmitri Sviridov, Benjamin T. Kile, Douglas J. Hilton

**Affiliations:** 1 Department of Biochemistry and Molecular Biology, Monash University, Melbourne, Australia; 2 Department of Anatomy and Developmental Biology, Monash University, Melbourne, Australia; 3 Division of Molecular Medicine, The Walter and Eliza Hall Institute of Medical Research, Melbourne, Australia; 4 Neonatal Services, Royal Women's Hospital, Melbourne, Australia; 5 Baker Heart Research Institute, Melbourne, Australia; 6 The Peter MacCallum Cancer Centre, St. Andrews Place, East Melbourne, Australia; 7 Division of Bioinformatics, The Walter and Eliza Hall Institute of Medical Research, Melbourne, Australia; 8 The Department of Medical Biology, The University of Melbourne, Melbourne, Australia; Harvard Medical School, United States of America

## Abstract

Harlequin Ichthyosis (HI) is a severe and often lethal hyperkeratotic skin disease caused by mutations in the ABCA12 transport protein. In keratinocytes, ABCA12 is thought to regulate the transfer of lipids into small intracellular trafficking vesicles known as lamellar bodies. However, the nature and scope of this regulation remains unclear. As part of an original recessive mouse ENU mutagenesis screen, we have identified and characterised an animal model of HI and showed that it displays many of the hallmarks of the disease including hyperkeratosis, loss of barrier function, and defects in lipid homeostasis. We have used this model to follow disease progression in utero and present evidence that loss of Abca12 function leads to premature differentiation of basal keratinocytes. A comprehensive analysis of lipid levels in mutant epidermis demonstrated profound defects in lipid homeostasis, illustrating for the first time the extent to which Abca12 plays a pivotal role in maintaining lipid balance in the skin. To further investigate the scope of Abca12's activity, we have utilised cells from the mutant mouse to ascribe direct transport functions to the protein and, in doing so, we demonstrate activities independent of its role in lamellar body function. These cells have severely impaired lipid efflux leading to intracellular accumulation of neutral lipids. Furthermore, we identify Abca12 as a mediator of Abca1-regulated cellular cholesterol efflux, a finding that may have significant implications for other diseases of lipid metabolism and homeostasis, including atherosclerosis.

## Author Summary

Harlequin Ichthyosis is a severe inherited disease in which the skin develops as thick armour-like plates. While many HI patients die at birth, those who survive are subject to dehydration and infection. The disease is caused by defects in a protein called ABCA12, which is thought to function by transporting lipids within the cells of the skin. Here, we describe a new genetic screen that we have used to identify a mouse model that develops the hallmarks of HI and carries a mutation in Abca12. We have used this model to elucidate Abca12's significant role in the transport of lipids within the skin, and we demonstrate that the loss of these lipids contributes to the dehydration in affected embryos and newborns. We attribute specific transport functions to the protein and show that it can mediate the efflux of a number of different lipids from the cell including, importantly, cholesterol. Cholesterol transport by proteins related to Abca12 plays a critical role in the development of a number of diseases, including heart and peripheral vascular disease, and the description of these functions for Abca12 suggest that it may play a wider role in controlling lipid metabolism.

## Introduction

Harlequin ichthyosis (HI, OMIM 242500) is a rare and devastating congenital disorder characterised by premature delivery and thick, hyperkeratotic, ‘armour’-like skin plaques. This immobile skin or ‘collodion membrane’ constricts the embryo causing odema, limb contractures and eversion of the eyelids and lips. Despite the provision of neonatal intensive care to ameliorate dehydration and the application of high-dose retinoid therapy [1], many infants die from respiratory distress, bacterial infections and feeding difficulties [2]. In surviving patients, the skin barrier dysfunction remains, leading to excessive transepidermal water loss, impairment of thermal regulation and an increased risk of cutaneous infection. The gross phenotypic and barrier defects in HI are thought to primarily result from abnormal lipid metabolism in the epidermis.

In mammalian skin the outer layer, or stratum corneum, maintains barrier function. Within this layer, corneocytes are embedded in a lamellar intercellular lipid complex of cholesterol, phospholipids and ceramides. Small, specialised vesicular structures known as lamellar bodies (LBs) are thought to traffic many of these components to the surface of differentiating keratinocytes [3]. Ceramides contribute to both lamellar extracellular lipids and to a covalently attached lipid layer known as the corneocyte lipid envelope (CLE) [4]. They are derived primarily from the conversion of glucosylceramides through the action of β-glucocerebrosidase [5] and to a lesser extend by the conversion of sphingomyelin by sphingomyelinase [6]. Most ceramide processing in the stratum corneum is thought to occur extracellularly after docking of the LBs with the cell surface, however significant levels of glucosylceramides and ceramides are found within the cell and in other layers of the epidermis [7].

Two independent studies have established that mutations in the *ATP binding cassette A12* (*ABCA12*) gene cause HI [8],[9]. The ABC proteins are thought to act primarily as transporters of molecules across cellular membranes and like other family members *ABCA12* encodes a polytopic transmembrane (TM) protein comprising at least 12 TM domains and 2 ATP binding cassettes. Mutations in *ABCA12* are also associated with a less severe disease known as lamellar ichthyosis-2 (LI2, OMIM 601277)[10]. Initial studies of these conditions indicate that LI2 is caused by missense, potentially hypomorphic, mutations in or near the first ATP binding domain (NBD1) whereas HI is associated with mutations that either abolish ABCA12 protein production or produce a protein with severely impaired function [8]–[10].

The co-localisation of ABCA12 with LBs [11], the common malformation of these organelles in HI [12], the mis-localisation of glucosylceramide in HI keratinocytes and the correction of this abnormality by ABCA12 expression [8] present prima facie evidence that the protein plays an active role in trafficking lipids into LBs. More specifically, the abnormal LBs in HI granular layer keratinocytes and lack of extra-cellular lipid lamellae in patients imply that lipid transport to the intercellular lamella is disrupted. Despite these observations the nature and scope of Abca12's involvement in lipid homeostasis remain unclear. Several of the 48 member ABC protein family are known to play critical roles in controlling lipid levels, primarily by mediating their efflux from the cell. Cholesterol metabolism is perhaps the best studied of these pathways, as defects in clearance of cholesterol from vascular cells constitute a key element in the development of atherosclerosis. ABCA1, in particular, is considered the primary mediator of cholesterol efflux and mutations in the gene are associated with reduced cholesterol efflux and absent reverse cholesterol transport in both humans (Tangier disease) and in animal models [13]–[15].

Genetic studies in the mouse have proven to be a very powerful approach to understanding human diseases that affect embryonic development. We have undertaken a genotype driven ENU screen which identifies pedigrees in which mice die embryonically or neonatally, irrespective of the cause or timing of death, and simultaneously maps the causative mutations within the genome. Using this strategy we have identified a pedigree carrying a mutation in one of the transmembrane domains of Abca12. Pups homozygous for the mutation die shortly after birth and show hallmarks of HI including hyperkeratosis, abnormal extracellular lipid lamellae and defects in cornified envelope processing. We have used this model to follow disease progression in utero and we report profound defects in lipid homeostasis demonstrating the extent to which Abca12 plays a pivotal role in maintaining the skin's lipid balance. Our study identifies Abca12 as a key regulator of lipid transport and homeostasis, and describes specific lipid efflux functions, including that of cholesterol, with broader implications for other lipid-related metabolic disorders.

## Results

### A Novel Recessive ENU Mutagenesis Screen Identifies an Animal Model of HI

**Figure 1:**
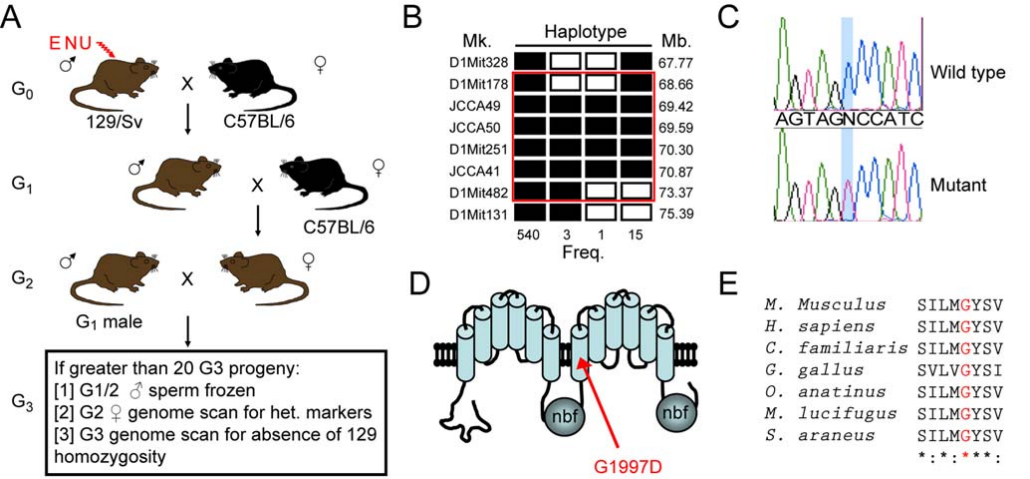
An ENU recessive mutagenesis screen identifies a lethal mutation *Abca12*. Mutagenised 129/Sv males were crossed with C57BL/6 females and the resultant G_1_ males crossed again to C57BL/6. Pedigrees were established by crossing G_1_ males with their G_2_ daughters and the G_3_ offspring were then subjected to genome wide screening for absence of homozygosity of the mutagenised (129/Sv) strain (A). The recessive *embryo lethal 12* (*el12*) mutation was identified using this approach and mapped by recombination to Chromosome 1 (B). Open rectangles indicate haplotypes homozygous for the 129/Sv mutagenised background and filled rectangles are C57BL6/J homozygotes or heterozygotes. Recombination frequency and markers position are indicated. A missense G1997D mutation was identified in *Abca12* (C, D). The *el12* mutation alters a residue in the second TM region of the protein which is conserved in all species examined (human, mouse, dog, chicken, platypus, microbat and shrew) (E).

Mutations that cause recessive lethality in embryos or neonates (and markers to which they are closely linked) are homozygous at reduced frequency among adults. This banality formed the basis of a genetic screen to identify genes required for mouse development (Figure 1A). Briefly, 129/Sv male mice were injected with ENU and mated to C57BL/6 females. Their first-generation (G_1_) male progeny were again crossed to C57BL/6 females, and then backcrossed to one of their second-generation (G_2_) daughters to yield a third-generation (G_3_). For those pedigrees in which 20 or more G_3_ mice were generated, the sperm of the founding G_1_ mouse was frozen. Adult G_3_ mice were genotyped with a panel of simple sequence length polymorphic markers and regions in which no animals showed homozygosity of the 129/SV alleles, despite both parents being heterozygous, were highlighted as being linked to a potential recessive lethal ENU-induced mutation. The presence of recessive lethal mutation was then confirmed by generating and genotyping a second cohort of G_3_ animals from the frozen sperm of the founding G_1_ male.

In our initial screen, we set up 40 G_1_ male mice to breed and generated 18 pedigrees that contained more than 20 G_3_ mice. To prove the principle of the approach, we have proceeded with one pedigree, *Embryonic Lethal 12* (EL12). In this pedigree, we found 129/SV alleles that were absent in all of the adult G_3_ mice. Notably, among 34 G_3_ EL12 mice, we observed none that were homozygous for the129/Sv allele of *D1Mit156*, even though both parents were heterozygous for the 129/Sv allele of this marker. This was confirmed in a second cohort of 31 G_3_ mice. Using a total of 463 mice and 13 polymorphic markers, we refined the interval harboring the lethal mutation to 4.7 Mb between D1Mit178 and D1Mit482 (Figure 1B). We sequenced the exons and intron/exon boundaries of the 13 genes in the candidate interval and found a single G to A transition of exon 41 of *Abca12* (Figure 1C). Abca12 is a member of the ABC transporter family of proteins, and the mutant allele (*Abca12^el12^*) results in a point mutation (G1997D) in the first helix of the protein's second transmembrane array (Figure 1D) which is highly conserved in a diverse range of organisms (Figure 1E).

### *Abca12^el12//el12^* Mice Die Neonatally

**Figure 2:**
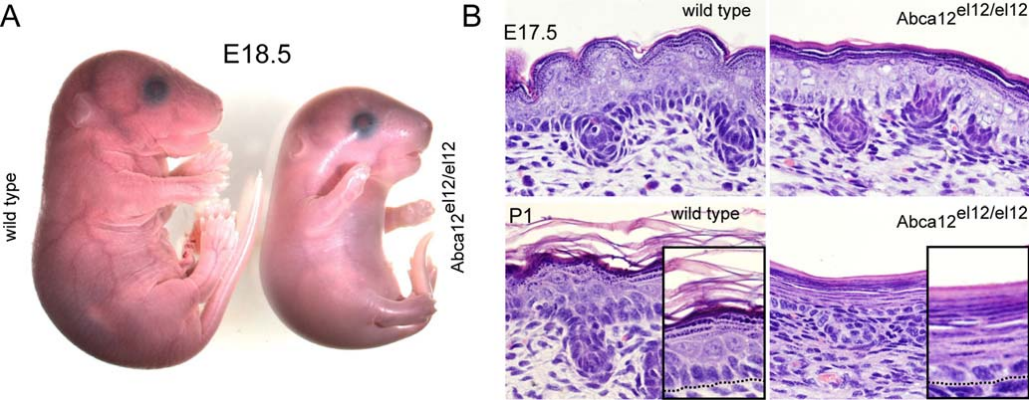
The *Abca12^el12/el12^* phenotype. *Abca12^el12/el12^* mice display an epidermal phenotype visible from E16.5 and by E18.5, the thickening of the cornified envelope produces a constrictive, taut and shiny epidermis resulting in limb contractures (A). Sections of epidermis at E17.5 and birth demonstrate severe hyperkeratosis characterised by the formation of a 20–30 cell layer thick stratum corneum. Cell architecture in other layers of the epidermis is also affected, with a reduction in the size of the spinous cell layer and lack of dense palisaded basal cell nuclear architecture (B).

Consistent with the results of the genetic screen, at weaning no *Abca12^el12/el12^* mice were detected from heterozygous crosses however examination of litters at E18.5 found normal mendelian ratios of viable but phenotypically abnormal *Abca12^el12/el12^* embryos (n = 17/57 embryos). *Abca12^el12/el12^* pups were occasionally found in the first few hours after birth but were often dead or severely dehydrated and had failed to suckle normally. Recent studies by Yanagi et al., indicate a role for Abca12 in lung development and defects in this organ may contribute to neonatal death [16]. To follow the development of the phenotype we examined cohorts of embryos from various developmental stages. At E14.5 and E15.5 homozygous embryos appeared normal; however from E16.5 onwards they were characterised by an absence of normal skin folds around the trunk and limbs. As development progressed, *Abca12^el12/el12^* embryos developed a taut, thick epidermis and multiple contractures affecting the limbs (Figure 2A,3A). Late stage *Abca12^el12/el12^* embryos were also found to be smaller than their wild type or heterozygous littermates (Figure 2A, 3A), a phenotype we assayed in newborn mice (p = 0.0023, data not shown). Skin sections from affected embryos revealed a hyperkeratotic phenotype from E16, and confirmed the absence of normal folding (Figure 2B).

**Figure 3:**
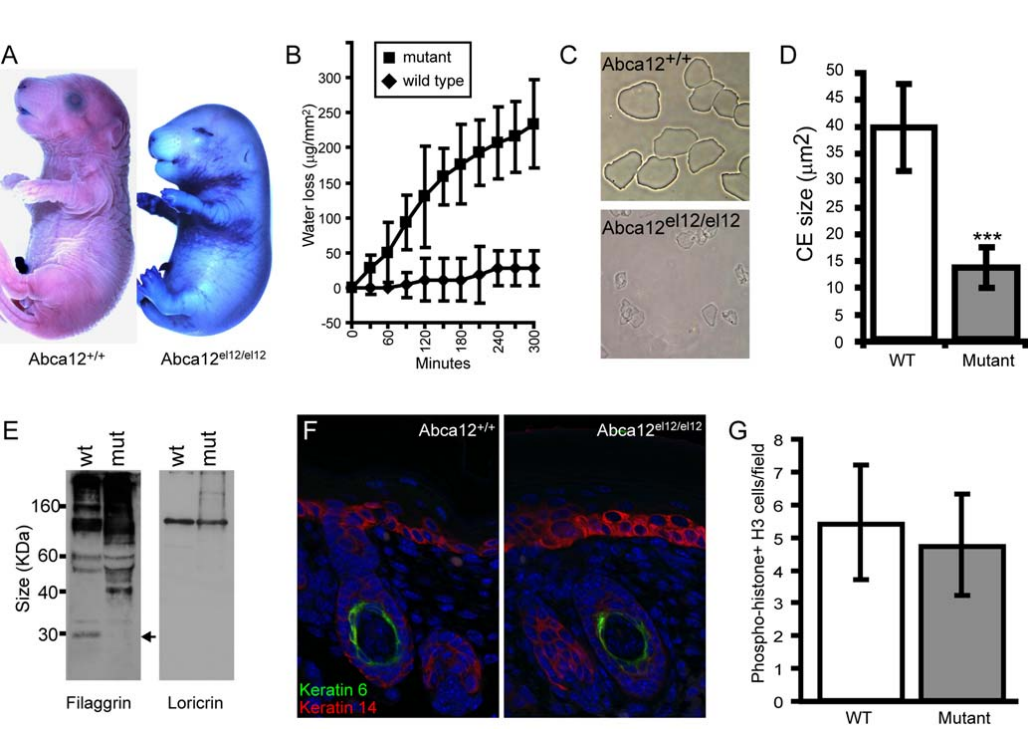
Barrier defects in *Abca12^el12/el12^* mice. *Abca12^el12/el12^* mice have defects in barrier formation as evidenced by dye exclusion (A) and trans-epidermal water loss (B) assays at E18.5. Cornified envelopes prepared from *Abca12^el12/el12^* mice are fragile and reduced in size compared with wild type littermate controls (*Abca12^el12/el12^* CEs concentrated 15 times, C, D: p = 6×10^−23^). Western blotting indicates defects in filaggrin processing in *Abca12^el12/el12^* epidermis (E, arrow) while expression of other CE proteins such as loricrin is unaffected. Expression of “proliferative” keratin VI is present only in the differentiating hair follicle of both mutant and wild type epidermis (F) and cell proliferation at E17.5 is normal as assayed by phospho-histone H3 staining (G; p = 0.27).

**Figure 4:**
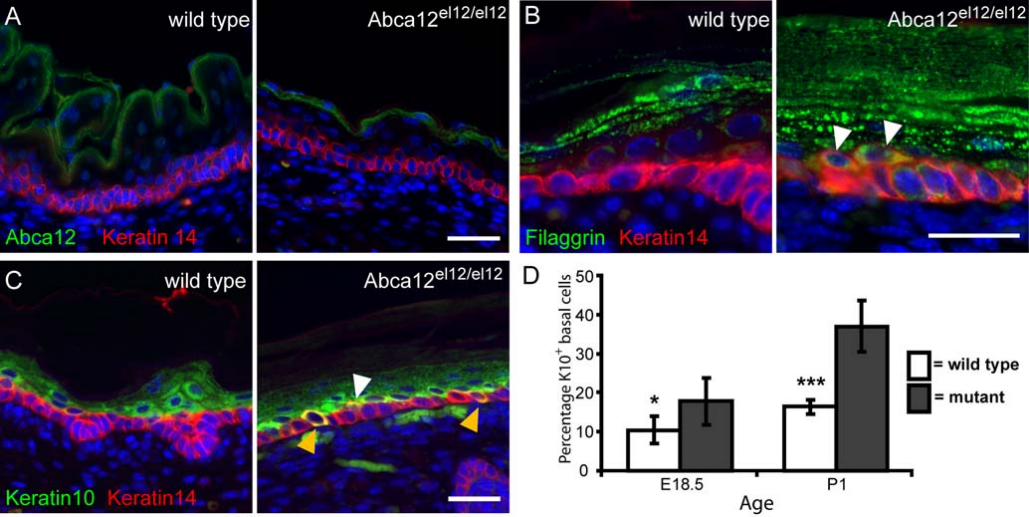
Pathology of the Abca12 mutant epidermis. Abca12 protein is detected in suprabasal keratinocytes in the granular and cornified cell layers of mutant and wild type epidermis (A). Keratinocytes in Abca12 mutant skin undergo premature differentiation highlighted by strong filaggrin expression in cells juxtaposed to K14^+^ basal keratinocytes (B) and increased co-expression of keratins 10 and 14 (white arrows), especially in basal cells (yellow arrows) (C). Increases in co-expression of K10 and K14 are significant at both E18.5 (p = 0.016) and at P1 (p = 4.44×10^−6^) (D). Samples are of E18.5 (A) or P1 (B, C) epidermis counterstained with DAPI. Scale bars = 30 µm.

Histologically all epidermal cell layers were apparent in *Abca12^el12/e1l2^* embryos, although the size of the granular layer progressively increased at the expense of the spinous layer (Figure 2B, 5A). By parturition the cornified layers had coalesced into thick sheets of 20–30 enucleate corneocytes. The basal layer in *Abca12^el12/el12^* mice also lost the dense palisaded nuclear organisation apparent in wild type and *Abca12^el12/+^* mice (Figures 2B, 4A). Consistent with the apparently restrictive nature of the cornified layer, the epidermis as a whole was 30% thinner at E17.5 and P1 in *Abca12^el12/el12^* animals (data not shown). Despite this constriction, hair follicles formed and differentiated relatively normally (Figure 2B, 3F) and complete histological examination of E18.5 embryos did not identify overt anomalies in other organs. Adult and embryonic *Abca12^el12/+^* mice had no overt phenotype, no obvious histological abnormalities and were healthy and fertile.

**Figure 5:**
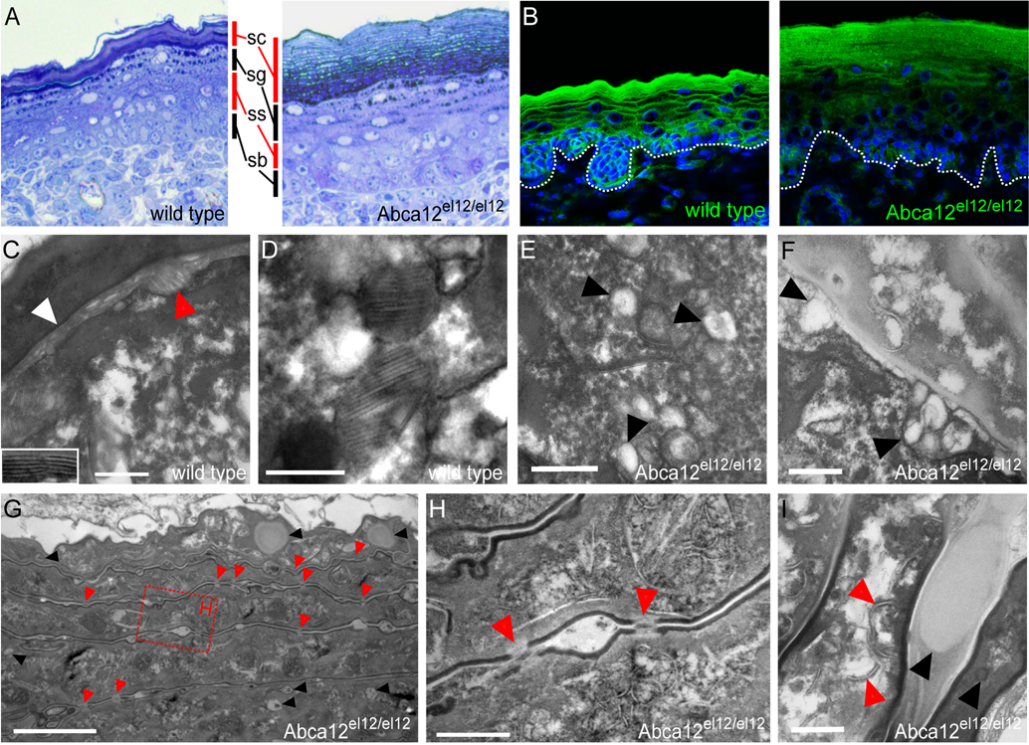
Ultrastructural defects in *Abca12^el12/el12^* mice. Thin sections of *Abca12^el12/el12^* epidermis illustrated hyperkeratosis and expansion of the stratum granulosum (A). Nile red staining shows reduced intercellular lamellae lipids at E18.5 (B). In wild type epidermis intercellular lipid lamellae (white arrow and inset) were noted as well as LBs fusing with the surface of granular cells (red arrow) (C). Lamellar bodies in wild type and heterozygous embryonic epidermis were normally loaded with lipid (D). In mutant skin, LBs lacked lamellar cargo (E, F arrowheads) but fused with the granular cell membrane (F; arrows). Mutant epidermis had a normal cornified envelope (G,H) with persistent corneodesmosomes in distal layers of the stratum corneum (G, H, red arrowheads) and the cornified layer had multiple lipid inclusions (G, black arrowheads). Unlike the uniform contents of wild type cornified cells mutant cell layers contained vesicular fibrillar structures (I, red arrows) and frequent inclusion bodies (I, black arrows). EM scale bars in C–F, H, I equal 200 nm and 2 µm in G. C–F and I were stained with ruthenium tetroxide, G and H with osmium tetroxide.

### *Abca12^el12/el12^* Mice Have a Defect in Skin Barrier Function

As the *Abca12^el12/el12^* mice apparently died from dehydration, we tested skin barrier function which normally initiates in the mouse from E16, and acquires almost full adult function by E18.5 [17]. We measured permeability of E18.5 embryos against the dye toluidine blue and found that *Abca12^el12/el12^* embryos had uniform absence of barrier function (Figure 3A). To determine if this defect contributed to the dehydration observed in homozygous animals we harvested the dorsal epidermis from E18.5 embryos and measured the ability of the skin to retain water using a trans-epidermal water loss (TEWL) assay over a 5 hour time course. A significant difference in TEWL from *Abca12^el12/el12^* embryos was observed as early as 60 minutes (Figure 3B), confirming that mutations in *Abca12* in mice also lead to the defects in barrier formation that are observed in HI patients.

### Defects in the Cornified Envelope of *Abca12^el12/el12^* Mice

HI patients develop a thick armour like stratum corneum and a suite of defects in the biochemistry of this layer. To examine the stratum corneum we harvested cornified envelopes (CE) from E18.5 embryonic skin. In wild type mice, large squames were present in expected numbers whereas *Abca12^el12/el12^* mice were found to have sparse CEs which were both small and unable to structurally withstand the purification procedure (Figure 3C,D). While the levels of filaggrin in the epidermis of E18.5 *Abca12^el12/el12^* mice were slightly increased, its processing into a functional 27 kDa monomer was ablated (Figure 3E) as has previously been observed in HI patients [18], indicating that normal LB and Abca12 function is required for this process. The level of other barrier proteins such as loricrin was unaffected (Figure 3E, data not shown). While keratin VI expression in interfollicular keratinocytes has been noted in some studies of HI skin [18], no aberrant expression of this hyperproliferative marker was detected in *Abca12^el12/el12^* mice (Figure 3F). These observations suggest that the hyperkeratotic phenotype in these animals is not a result of increased cell proliferation in the basal epidermal layer. To confirm these findings we surveyed cell proliferation and apoptosis from E17.5 to P1 (using Ki67, PCNA, phospho-histone H3 and TUNEL staining) and found no significant differences (Figure 3G, data not shown).

### *Abca12^el12/el12^* Epidermis Undergoes Premature Differentiation

Many defects of barrier function have profound impacts on the epidermis as a whole. We were able to show by histology that alterations in both nuclear organisation and cellular architecture of the basal cell layer characterises *Abca12^el12/el12^* embryos and postpartum epidermis. We investigated expression of markers of basal and differentiating keratinocytes during this period and observed normal levels of Abca12 staining in epidermal cells in the uppermost granular and cornified layers of the epidermis (Figure 4A), in a pattern similar to that observed in developing human skin [19]. Expression of filaggrin, which usually marks the granular layer of the epidermis, was detected in keratinocytes juxtaposed to the basal layer itself and in some cells expressing keratin 14 (Figure 4B). Additionally, we demonstrated a significant increase in basal (and spinous) layer keratinocytes dually expressing keratins 10 and 14 (Figure 4C,D). These observations indicate that keratinocytes in affected epidermis undergo premature differentiation, either as a result of defects in the cornifying layer which overlies them or as a consequence of defects in the balance of intracellular lipids in these cells.

### *Abca12^el12/el12^* Keratinocytes Have LB Defects

Thin sections of affected epidermis highlighted the striking hyperkeratosis in *Abca12^el12/el12^* epidermis (Figure 5A). To investigate Abca12 mediated alterations in epidermal lipid composition we stained the epidermis with the lipophilic dye Nile Red. *Abca12^el12/el12^* mice displayed very little of the normal lipid deposition in intercellular spaces of the cornified envelope (Figure 5B). To confirm these effects at an ultrastructural level we performed transmission electron microscopy on epidermal tissue at E18.5, utilising ruthenium tetroxide postfixation of thin epidermal sections [20] to investigate the lamellar lipids which normally surround cells of the stratum corneum. We demonstrated an absence of these elements in the spaces between the corneocytes and cornified/granular layer (Figures 5C–F), although the CLE was still apparent in *Abca12^el12/el12^* tissues, an observation previously observed in HI biopsies [21] (data not shown). The absence, relative scarcity or malformation of LBs is particularly characteristic of HI [18], [22]–[25]. While relatively scarce structures resembling LBs were apparent within the granular layers in *Abca12^el12/el12^* animals (Figure 5E,F), most lacked the multilayered lamellar cargos present in control skin (Figure 5D). Fusion of LBs with the surface of the normal granular cells was commonly observed in wild type skin (Figure 5C) and occurred occasionally in affected epidermis (Figure 5F). Normally, corneocytes are filled with uniformly opaque keratin-filaggrin protein, however in affected epidermis they contained numerous vesicular and lamellar structures (Figure 5H,I), defects found in both *in situ* and reconstituted HI epidermis [19],[21],[26]. Whilst our cellular and biochemical analysis suggested that some aspects of cornified cell envelope formation were disrupted in homozygous mice we did not observe overt differences in this structure during our EM studies. Indeed EM studies show that as well as a normally formed cornified envelope (Figure 5G–I) there is increased retention of corneodesmosomes in the distal layers of the stratum corneum (Figure 5G,H). The persistence of these structures provide a mechanistic basis for the hyperkeratotic phenotype in our animals and serves to explain the relative decrease in extraction of CE's as noted above. The epidermis of the *Abca12* homozygous mice bear many if not all of the features of HI, establishing them as an excellent model in which to study the biochemical basis of this disease.

### Defects in Lipid Homeostasis in *Abca12^el12/el12^* Epidermis

**Figure 6:**
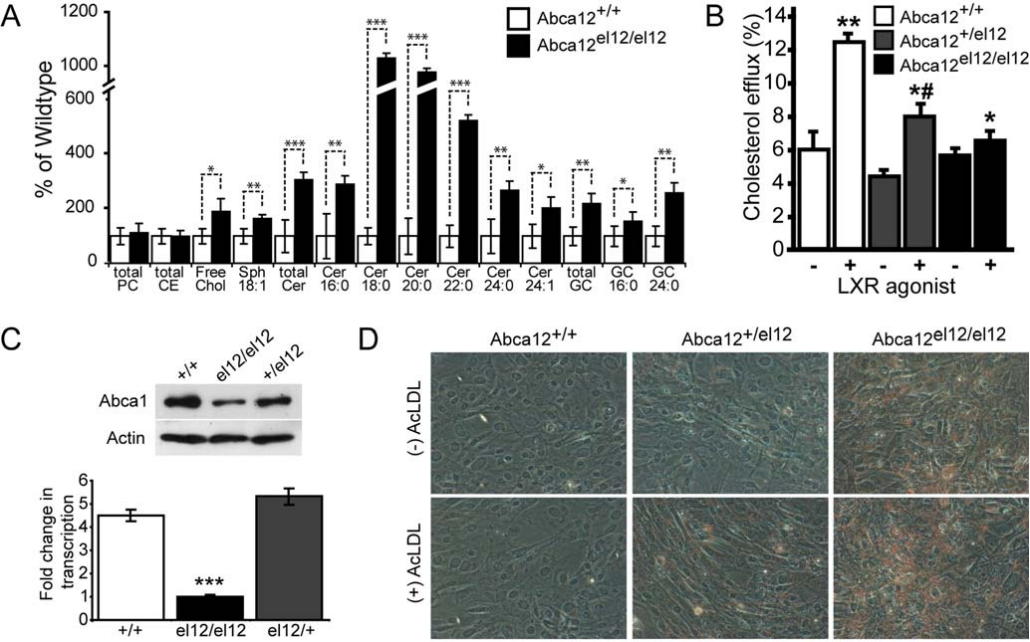
Defects in skin lipid composition and cellular lipid efflux mediated by Abca12. Analysis *Abca12^el12/el12^* epidermal lipids indicate significant increases in levels of ceramide (Cer), glucoslyceramide (GC), sphingosine (Sph) and free cholesterol in *Abca12^el12/el12^* skin at E18.5. Total phosophatidylcholine (PC) and cholesterol ester (CE) levels were unchanged (A, ***p<0.0001; **p<0.001; *p<0.01 versus wilt type epidermis) (A). Defects in fibroblast cholesterol efflux were assayed using [^3^H]cholesterol and incubation in the presence (+) or absence (−)) of the LXR agonist TO-901317. Means plus or minus standard deviation of quadruplicate determinations are shown. (**p<0.001 versus non activated cells; ^*#^ p<0.001 versus ABCA12^+/+^ cells; ^*^p<0.01 versus ABCA12^−/+^ cells) (B). Expression of Abca1 was determined at the protein level by Western blotting (C, upper panel) and decreases in *Abca12* homozygous mutant cells were shown to be in part due to decreases in transcription of *Abca1* (C, lower panel, fold change in transcription relative to el12/el12, ***p<0.0005). Lipid accumulation in fibroblasts the presence or absence of the lipid donor acetylated LDL (AcLDL) was assayed by Oil Red O staining (magnification ×100) (D).

While previous studies of cultured human HI keratinocytes have identified defects in the traffic of glucosylceramides, global analysis of defects in lipid homeostasis, either *in vitro* or *in vivo*, have not been performed. We therefore utilised the *Abca12^el12^* model to examine whether defects in lipid homeostasis were apparent in our mice. We harvested whole epidermis from the mid-dorsum of E18.5 homozygous, heterozygous and wild type littermates and empirically assayed for levels of a panel of thirteen different lipid species. Consistent with reports that Abca12 regulates the trafficking of glucosylceramide we detected greater than 2-fold increases in this lipid species in *Abca12^el12/el12^* epidermis (Figure 6A). We also detected striking increases in the relative levels of all ceramide species in affected versus wild type skin with highest proportional differences in C18, C20 and C22 species, indicating that their transport is also reliant on Abca12 function (Figure 6A). Sphingosine, a breakdown product of ceramide, was also markedly increased. This was in spite of the fact that we were unable to resolve intercellular lipid lamellae in the cornified envelope indicating an intracellular build-up of these species. Furthermore, significantly increased levels of cholesterol were observed in the epidermis of *Abca12^el12/el12^* mice (Figure 6A). No differences were observed in total levels of phosphatidylinositols, phosphatidylethanolamines, phosphatidylcholines, acyl- and lyso-phosphatidylcholines and sphingomyelin, suggesting that the defects apparent in our mice, while more broad and widespread than previously appreciated, were not a consequence of universal dysregulation of lipid homeostasis. Notably, we failed to identify any significant differences in lipid levels in *Abca12^el12/+^* skin, confirming the absence of a haplo-insufficient phenotype highlighted by our histological survey.

### Defects in Lipid Efflux in *Abca12^el12/el12^* Fibroblasts

Having established a role for Abca12 in lipid metabolism in keratinocytes we wondered whether the protein might be more widely involved in this process. To assess the generality of the involvement of Abca12 in lipid metabolism, we investigated lipid efflux from *Abca12^el12/el12^*, *Abca12^+/el12^* and *Abca12^+/+^* mouse skin fibroblasts [27]. To establish the specific involvement of ABC transporters, cholesterol efflux was compared with or without activation of LXR, which greatly increases expression of most ABC transporters including Abca12 [28]. As expected, in wild type cells activation of LXR resulted in a more than doubling of cholesterol efflux to apolipoprotein A-I (apoA-I) (Figure 6B). In *Abca12^+/el12^* cells the effect was less pronounced, but there was still a statistically significant increase of the efflux from activated versus non-activated cells. In contrast, in *Abca12^el12/el12^* cells activation of LXR did not result in elevation of cholesterol efflux. Specific, ABC-dependent cholesterol efflux (i.e. the difference in the efflux with and without activation) was virtually zero (Figure 6B). Current models suggest that the cholesterol efflux to apoA-I is fully controlled by ABCA1 [29]; however, in *Abca12^el12/el12^* cells there was no ABC-dependent efflux to apoA-I despite the animals having functional Abca1. Further, when phospholipid efflux was compared in *Abca12^el12/el12^* and *Abca12^+/+^* fibroblasts, activation of cells with LXR agonist resulted in a 25% increase in phospholipid efflux in *Abca12^+/+^* cells (p<0.05), but no increase in *Abca12^el12/el12^* (not shown). To determine whether the loss of Abca12 was affecting the production and abundance of the Abca1 protein in cells from *Abca12^el12/el12^* mice we performed western blotting for Abca1. Strikingly, loss of Abca12, even in a heterozygous state, led to concomitant decreases in Abca1 protein, providing a functional link between loss of Abca12 and impairment of cholesterol efflux (Figure 6C, upper panel). Analysis of transcription of *Abca1* in these cells highlighted 5 fold less expression in mutant versus wild type fibroblasts but no significant difference between wild type and heterozygotes (Figure 6C, lower panel).

Impairment of cholesterol efflux is a frequent cause of excessive accumulation of neutral lipids in cells, especially when exposed to acetylated low density lipoprotein (AcLDL), a cholesterol donor for poorly regulated cholesterol uptake pathways. We compared accumulation of neutral lipids in *Abca12^el12/el12^*, *Abca12^+/el12^* and *Abca12^+/+^* fibroblasts treated or not treated with AcLDL by staining lipids with Oil Red O. Wild type fibroblasts did not accumulate lipids independently of the presence of AcLDL indicating that lipid homeostasis pathways successfully cope with excessive lipid delivery (Figure 6D). *Abca12^+/el12^* fibroblasts also did not accumulate lipids in the absence of AcLDL, but there was visible lipid accumulation in the presence of AcLDL. *Abca12^el12/el12^*cells accumulated lipids both in the absence and presence of AcLDL, the accumulation being more severe in the presence of AcLDL. Thus, lipid homeostasis is severely impaired in *Abca12^el12/el12^* fibroblasts.

## Discussion

Forward genetic screens in mice remain an important source of models of genetic disorders in humans. In this report we have used a forward genetic approach to identify a model of harlequin ichthyosis which has allowed us to characterise Abca12's function as a key regulator of lipid homeostasis and cholesterol transport. Current recessive ENU mutagenesis approaches to identify embryonic lethal mutations in the mouse either require the analysis of large numbers of embryos to identify defects, or the use of mice carrying engineered balancer chromosomal rearrangements tagged with visible phenotypic markers [30]. While the latter approach can very efficiently identify all the mutations that cause lethality between conception and weaning, and has the advantage of simultaneously isolating and mapping mutations, the genomic region screened is restricted to that delimited by the balancer chromosome. We have developed a simple genome-wide approach which obviates the requirement to dissect embryos and which simultaneously isolates and maps mutations. We inter-crossed two inbred mouse strains, one of which was mutagenised with ENU, established pedigrees from the resultant offspring, and screened these for regions of the genome under-represented for the mutagenised genetic background. As a consequence we simultaneously identified and mapped lethal mutations in an unbiased genome wide manner.

Using this approach we have isolated a mouse model of Harlequin Ichthyosis, a hyperkeratotic and often lethal disease of the epidermis. We observed many HI features in our *Abca12^el12/el12^* mice including severe hyperkeratosis, LB defects, absence of intercellular lamellae, aberrant filaggrin processing, neonatal death, defects in lipid metabolism, congenital contractures and the absence of skin barrier function. Studies of HI pathology suggest that the disease may be grouped into 3 subtypes [18]. The altered LB structure, absence of keratin VI expression and defects in filaggrin processing indicate that our mutant is equivalent to Type 1 HI proposed by this scheme although recent genotype/phenotype analysis suggests no correlation between mutation and phenotype [31]. Defects in the CE are characteristic of LI [32],[33], but our EM investigation showed no obvious deficiencies in this structure. A missense mutation similar to that of our mouse (glycine to charged amino acid in a highly conserved TM domain residue) has been shown to cause severe HI [31]. It is highly unlikely that our model is of LI2, in which missense mutations have only been found within or near the first nucleotide binding domain of the protein [10]. This, coupled with the severity of disease in our mouse, suggests the G1997D mutation severely affects Abca12 activity although it remains to be determined whether it mis-localises or has altered transport function, as both have been observed in TM mutations in ABC family members causing severe disease [34]–[36]. The *Abca12^el12/el12^* phenotype closely matches a targeted deletion of exon 10 generated by Lexicon Genetics, an allele in which postnatal lethality and absence of heterozygous effects were noted (*Abca12^tm1Lex^*, www.informatics.jax.org). However, the Lexicon study undertook no characterisation of homozygous animals beyond noting lethality. A recent similar study by Yanagi et al., demonstrated barrier defects in mice lacking Abca12 and suggest that postnatal death is a result of defects in lung function in newborn animals [16]. Our mouse parallels HI in almost every respect and has allowed us to investigate several aspects of disease which have been impossible in the limited patient samples available.

We first examined the temporal progress of disease. *Abca12^el12/el12^* mice displayed severe hyperkeratosis around the time of stratification of the cornified epidermal layer (E16.5). This phenotype increased in severity as development progressed to the point where the epidermis restricts the normal growth of the embryo. *Abca12^el12/el12^* skin progressively enters a state of premature differentiation characterised by loss of normal basal cell architecture, mis-expression of differentiated keratins in basal keratinocytes, reduction in the size of the spinous cell layer and expansion of granular layers. We show that the retention of cornified squames in the upper layers of the epidermis which contribute to this restrictive epidermis is not due to hyper-proliferation or alteration in apoptosis. Instead we observe defects in the deposition of extra-cellular lipid lamellae and in proteolytic activity in the epidermis, indicating that the hyperkeratosis in our mice is due to failure to form and shed cornified envelopes from the skin surface. Our EM studies indicated that this retention was in part due to persistence of corneodesmosomes into the distal layers of the epidermis. This retention defect may explain why the CE's isolated directly from the skin surface by detergent extraction were relatively sparse and also exhibit fragility. Our results are consistent with previous studies indicating that the defects in LB loading can result in decreases in co-transport of proteases required for normal desquamation [37] and which has been suggested as a mechanism by which HI hyperkeratosis might occur [38]. Our results lend weight to this hypothesis. These defects also contribute to the loss of barrier function of mutant epidermis. As with human HI patients [18], defects in proteolytic cleavage of filaggrin characterise the mice. These defects in the proteolytic processing of components are almost certainly reflected in the unusual presence of inclusions and vesicles within the normally uniform cells of the stratum corneum when examined by EM. In addition to these defects in the cornified layer, our observation of differentiation defects in *Abca12^el12/el12^* mice indicates that defects in the HI epidermis affect all layers of the skin.

Insights into the mechanisms by which loss of Abca12 function might affect the skin was revealed by our analysis of lipid species present in the epidermis. Previous studies have shown that Abca12 is important in controlling glucosylceramide trafficking in keratinocytes [8], where it localises to the golgi and lamellar bodies [11], an observation which correlates well with the striking increase in levels of glucosylceramide in the *Abca12^el12/el2^* epidermis. However, our investigations of the *Abca12^el12/el12^* mice revealed that defects in lipid homeostasis in the skin extend well beyond glucosylceramide. Despite the absence of intercellular lipid lamellae we detected significant increases in both ceramide and free cholesterol in the epidermis. We propose that increases in ceramide (and indeed sphingosine, a ceramide breakdown product) might reflect continuing unchecked de-novo synthesis and accumulation of this species, because of the absence of an Abca12 mediated trafficking mechanism to remove glucosylceramide from the cell. Cholesterol is also a known cargo of lamellar bodies [3] and its increased concentration in the epidermis probably reflects defects in trafficking of LBs or of a failure of loading this component as a consequence of loss of Abca12. The ratio of ceramide, cholesterol and fatty acids in the epidermis is also a key determinant of barrier function in the skin, and normal LB formation [39] and induction of the synthesis machinery for these compounds is an early response to compromises in barrier function [40],[41]. Consequently the defects in lipid levels in Abca12 mutant skin might actually be exacerbated by these positive feedback loops beyond primary defects related to Abca12 transport dysfunction.

We find that the effects of Abca12 mutation are not limited to keratinocytes. Skin fibroblasts isolated from mutant mice showed an impairment of their ability to maintain cholesterol efflux to apoA-I proportional to gene dose. Cholesterol efflux to apoA-I is a key pathway responsible for maintaining cellular cholesterol homeostasis and is believed to be fully controlled by another ABC transporter, ABCA1 [42]. Here we demonstrate that this is not the case, and that Abca12 is also essential for cholesterol efflux to apoA-I. Phospholipid efflux was also impaired, consistent with the currently adopted view of the mechanism of ABCA1-dependent cholesterol efflux [43]. We demonstrate that in primary cells from *Abca12^el12/el12^* mice, loss of Abca12 function results in decreased transcription of *Abca1*. The basis of this association remains unclear but the alteration in transcription may not be the only explanation of significant ablation of ABCA1-dependent cholesterol efflux observed in these cells. This is particularly notable in fibroblasts heterozygous for the el12 mutation, which have normal levels of *Abca1* transcription but which display significant decreases in Abca1 abundance and in efflux of cholesterol to apoA-1. Given that many ABC transporters form homo- or hetero-oligomers and that oligomerisation of Abca1 is important for its function [44] we speculate that a direct association between Abca12 and Abca1 might additionally be essential for functional stabilization of Abca1 and normal cholesterol efflux to ApoA1. The exact mechanism by which this occurs remains to be determined. Significantly, fibroblasts lack the classic LB organelles observed in keratinocytes. Our results therefore indicate that the Abca12 protein is capable of regulating the accumulation and efflux of lipids without this highly specialized organelle.

Impairment of cholesterol efflux led to intracellular accumulation of neutral lipids, most likely cholesteryl esters; in *Abca12^el12/el12^* cells. These lipids accumulated even in the absence of a challenge with extra-cellular lipid delivery through AcLDL. This finding has implications for another important pathology, atherosclerosis. Accumulation of cholesterol is a crucial element of the pathogenesis of atherosclerosis and impairment of cholesterol efflux, especially against a background of hypercholesterolemia, is a key contributor to the risk of atherosclerosis and coronary heart disease. Polymorphisms in *ABCA1* are one of the strongest factors affecting plasma levels of high density lipoprotein [29] and risk of cardiovascular disease [45]. Our findings suggest that Abca12 is also required for the cholesterol efflux pathway to function and therefore should be taken into account when investigating mechanisms of atherosclerosis or considering targets for its treatment. *Abca12* is expressed in primary macrophages at levels approximately 10 fold greater than the fibroblasts in which we have demonstrated cholesterol efflux defects in this study (unpublished observations). The severity and rarity of HI have precluded studies addressing associations between HI and heart disease, but the availability of this mouse model will allow us to investigate this relationship.

We have detailed a genetic screening protocol which concurrently identifies and maps postnatal or embryonic lethal mutations in an unbiased genome wide manner. This approach has allowed us to characterise an animal model of HI, providing a unique avenue by which to pursue therapeutic interventions for this and other ichthyoses. Our results demonstrate that HI should be viewed as a disease in which defects in Abca12 function lead to profound dysregulation of lipid metabolism in the epidermis. Furthermore we show in fibroblasts that the protein is a key regulator of cholesterol efflux, an observation with direct relevance to other defects of lipid homeostasis, including atherosclerosis.

## Materials and Methods

### ENU Mutagenesis Screen

Male 129/Sv mice were injected with a total dose of 200–400 mg/kg of N-Ethyl-N-Nitrosourea (ENU) in 3 weekly doses. ENU-treated males were mated with a C57BL/6 female and male G_1_ mice were crossed to C57BL/6 females. A G_2_ daughter was then backcrossed to her G_1_ father to produce G_3_ progeny for typing using 139 polymorphic markers spaced evenly throughout the genome [46]. Genotyping the G_2_ female allowed us to identify those markers that were heterozygous and hence informative in the final screen of G_3_ mice. Embryonic lethal mutations therefore manifest as a reduction in the expected frequency of 129/Sv homozygosity in adult animals. Those pedigrees in which no 129/Sv homozygosity of an informative SSLP was observed in G_3_ mice at weaning were recovered by performing IVF using cryopreserved G_1_ male sperm and eggs from C57BL/6 females. G_2_ mice heterozygous for the region of interest were then inter-crossed and their progeny analysed to determine whether 129/Sv homozygosity of linked markers was also absent in the second cohort. In these cases, the location of the embryonic lethal mutation was refined by genotyping key recombinants with additional polymorphic markers. Genomic DNA was extracted from tail biopsies and subjected to PCR amplification with oligonucleotide primers designed using the GABOS/GAFEP program (http://bioinf.wehi.edu.au/gabos/index.php). To remove primers and unincorporated nucleotides, post-PCR reactions were treated with ExoSAP-IT (USB) according to the manufacturer's instructions and filtered through Sephadex columns. Amplicons were then sequenced directly using BigDye Terminator v3.0 (Applied Biosystems).

### TEWL and Skin Permeability Assays

Assays of epidermal barrier function were performed essentially as previously described [17]. Gravimetric TEWL assays were performed using skin samples excised from the lateral thoracolumbar region of E18.5 embryos. Embryos and skin were photographed with a Zeiss Axiocam camera mounted on a Zeiss Stemi microscope. Comparison of TEWL was made using logistic regression models. All other statistical analyses were performed using the statistical software package STATA Version 7 (Stata Corporation USA).

### Cornified Envelope Preparations

Cornified envelopes and epidermal protein samples were prepared as previously described [47],[48]. Size calculations were performed using the Image J software package (NIH).

### Immunohistochemistry

Nile Red staining was performed as previously described [49]. IHC and IF were performed on citrate antigen retrieved paraffin embedded tissues or on frozen sections. Antibodies used were: rabbit anti-cytokeratin 14, -cytotkeratin 10, -cytokeratin 6, -filaggrin and -loricrin (Covance, 1∶1000); mouse anti-keratin 14 (1∶200, LL002, gift from Fiona Watt); goat anti-Abca12 (Santa Cruz Biotechnology, 1∶50). Secondary antibodies were from Molecular Probes. Samples were imaged by epifluorescence on an Olympus Provis AX70 or by confocal, using a Lecia SPE microscope. Cell proliferation and differentiation were assayed by counting phospho-histone H3^+^ and K14^+^/K10^+^ positive basal interfollicular keratinocytes in multiple fields under 20× magnification (n = 15 and 7 respectively).

### Transmission Electron Microscopy

Mid-dorsum skin was processed for EM as described [20] with minor modifications. Following fixation and cryoprotection, samples were OCT embedded, frozen on dry ice and 40 µm sections cut using a Leica CM 3050 S cryostat. Washed samples were post-fixed with 0.2% ruthenium tetroxide (Polysciences, USA), 0.5% potassium ferrocyanide in 0.1 M sodium cacodylate, pH 7.4 in the dark for 60 min. Following rinsing in water, samples were dehydrated in an alcohol series and embedded in Spurrs resin. Sections were cut using a Leica Ultracut S ultra-microtome, mounted on copper grids and contrasted with methanolic uranyl acetate and aqueous lead citrate before imaging in a JEOL 1011 TEM with a MegaView III CCD cooled digital camera (Soft Imaging Systems, Germany).

### Whole Epidermis Lipid Analysis

All solvents were of HPLC grade and were used without further purification. N-Palmitoyl-*d3*-glucosylceramide (GC 16:0(*d3*)) and N-palmitoyl-*d3*-lactosylceramide (LC 16:0(*d3*)) were from Matreya Inc. (Pleasant Gap, USA). Sphingosine (Sph, 17:1 base), ceramide (Cer) 17:0, sphingomyelin (SM)16:0*(d31)* and phosphatidylcholine (PC) 17:0/17:0 were from Avanti Polar Lipids (Alabaster, USA), Cholesteryl ester (CE) 17:0 was from Mp Biomedicals (Seven Hills, NSW, Australia). Lipid analysis was performed independently on 7 *Abca12^+/+^*, 8 *Abca12^el12/+^* and 6 *Abca12^el12/el12^* embryos. E18.5 fetus skins were incubated in phosphate buffered saline containing 5 mM EDTA for 1 h at 37°C. The epidermal layer was then peeled from the skin with tweezers, weighed and homogenized in 1.0 ml of PBS using a dounce homogenizer. Protein determination was performed using the Micro BCA Protein Assay Kit (Pierce, Rockford, Il, USA). Total cholesterol was determined using the Amplex Red Cholesterol Assay Kit (Invitrogen, Mount Waverly, Vic, Australia). Total lipids were extracted from tissue homogenates (100 µL containing approximately 100 µg protein) according to established methods [50], incorporating 400 pmol of each of the following internal standards: GC 16:0(*d3*), LC 16:0(*d3*) Sph (17:1 base), Cer 17:0, SM16:0*(d31)*, PC 14:0/14:0 and CE 17:0. Lipid extracts were reconstituted in 200 µL 10 mM, NH_4_COOH in methanol. Lipid analysis was performed by liquid chromatography, electrospray ionisation-tandem mass spectrometry (LC ESI-MS/MS) using a HP 1100 liquid chromatography system combined with a PE Sciex API 2000 Q/TRAP mass spectrometer with a turbo-ionspray source (250°C) and Analyst 1.4.2 data system. LC separation of lipids was performed on an Alltima C18, 3 um, 50×2.1 mm column using the following gradient conditions; 70% A reducing to 0% A over three minutes followed by 5 minutes at 0% A, a return to 70% A over 0.1 minute then 1.9 minutes at 70% A prior to the next injection. Solvent A and B consisted of tetrahydrofuran∶methanol∶water in the ratios (30∶20∶50) and (70∶20∶10) respectively, both containing 10 mM NH_4_COOH. Quantification of individual species of Sph, Cer, GC, LC, SM, PC and CE was performed using multiple-reaction monitoring (MRM) in positive ion mode. MRM product ions used were *m/z* 264 [sphingosine–H_2_O]^+^ for sphingosine, Cer, GC and DHC, *m/z* 184 [phosphocholine]^+^ for SM, PC and *m/z* 369 [cholesterol-H_2_O]^+^ for CE. Each ion pair was monitored for 50 ms with a resolution of 0.7 amu at half-peak height and averaged from continuous scans over the elution period. Lipid concentrations were calculated by relating the peak area of each species to the peak area of the corresponding internal standard.

### Isolation of Embryonic Skin Fibroblasts

The skin was separated from the mouse embryos (last week of gestation). Skin tissue was finely minced, resuspended in 0.05% Trypsin/EDTA solution, incubated for 30 min at 37°C, vigorously shaken, incubated for another 10 min at 37°C and neutralized with medium containing 10% FBS. Cells were seeded and incubated overnight in CO_2_ incubator before unattached cells and debris were washed out.

### qPCR Analysis of Abca1 Expression

Quantitative expression of *Abca1* was determined by qPCR from cDNA transcribed from Trizol prepared sample total RNA and amplification using SYBR GreenER PCR mix (Invitrogen) by primer sequences previously optimized for this approach [51]. Assays were performed in triplicate and standardized to an internal 18S rRNA control.

### Lipid Efflux Assays

Human HDL (1.085<d<1.21) and apoA-I were isolated from pooled normolipidemic human plasma supplied by Red Cross as described previously [52]. LDL was purified from human plasma by sequential centrifugation and acetylated as described by Basu et al. [53]. Cholesterol and phospholipid efflux were assessed as described previously [54]. Briefly, fibroblasts were incubated in labeling medium containing [^3^H]cholesterol (75 kBq/ml) or [methyl- ^14^C] choline (0.2 MBq/ml) for 48 hours. Cells were then incubated for 18 hr in serum-free medium in the presence or absence of the LXR agonist TO-901317 (final concentration 4 µM) to stimulate expression of ABC transporters and cholesterol efflux. Cells were then washed with PBS and incubated for 2 h in either serum-free medium alone (blank) or in serum-free medium supplemented with 30 µg/ml of lipid-free apoA-I. For cholesterol efflux analysis, aliquots of medium and cells were counted. For phospholipid efflux lipids were extracted from cells and medium [50] and counted. The efflux was calculated as radioactivity in the medium/(radioactivity in the medium+radioactivity remaining in the cells)×100%. Non-specific efflux (i.e. the efflux in the absence of acceptor) was subtracted.

### Oil Red O Staining

Cells were incubated in the presence of TO-901317 (final concentration 4 µM) and in the presence or absence of AcLDL (10 µg/ml) in serum-containing medium for 18 hrs. After washing with PBS, cells were fixed in 3.7% formaldehyde for 2 min, washed with water, and incubated at room temperature for 1 h with Oil Red O working solution (Fisher Biotech).
