# Correction: A Mouse Model of Harlequin Ichthyosis Delineates a Key Role for Abca12 in Lipid Homeostasis

**DOI:** 10.1371/annotation/02a793a0-160f-46b9-abaa-4a3d3eecdde2

**Published:** 2008-10-31

**Authors:** Ian Smyth, Douglas F. Hacking, Adrienne A. Hilton, Nigora Mukhamedova, Peter J. Meikle, Sarah Ellis, Keith Slatterley, Janelle E. Collinge, Carolyn A. de Graaf, Melanie Bahlo, Dmitri Sviridov, Benjamin T. Kile, Douglas J. Hilton

The seventh author's name was spelled incorrectly. The correct name is: Keith Satterley.

